# Nebivolol Ameliorates Cardiac NLRP3 Inflammasome Activation in a Juvenile-Adolescent Animal Model of Diet-Induced Obesity

**DOI:** 10.1038/srep34326

**Published:** 2016-09-30

**Authors:** Qihai Xie, Tong Wei, Chenglin Huang, Penghao Liu, Mengwei Sun, Weili Shen, Pingjin Gao

**Affiliations:** 1 State Key Laboratory of Medical Genomics, Shanghai Key Laboratory of Hypertension, Department of Hypertension Ruijin Hospital, Shanghai Jiaotong University School of Medicine, Shanghai, China; 2 Department of Cardiology, Shanghai Jiading District Central Hospital, Shanghai, China; 3 Key Laboratory of State General Administration of Sport, Shanghai Research Institute of Sports Science, Shanghai, China

## Abstract

NLRP3 is involved in obesity-induced cardiac remodeling and dysfunction. In this study, we evaluated whether the cardiac protective effects of nebivolol relied on attenuating NLRP3 activation in a juvenile-adolescent animal model of diet-induced obesity. Weaning male Sprague-Dawley rats were fed with either a standard chow diet (ND) or a high-fat diet (HFD) for 8 weeks. The obese rats were subsequently subdivided into three groups: 1) HFD control group; 2) HFD with low-dose nebivolol (5 mg/kg/d); 3) HFD with high-dose nebivolol (10 mg/kg/d). Treatment with nebivolol prevented HFD-induced obesity associated excess cardiac lipid accumulation as well as myocardial mitochondrial dysfunction. Nebivolol attenuated pro-inflammatory cytokines secretion and NLRP3 inflammasome activation in myocardium of obese rats. In parallel, nebivolol treatment of obese animals increased cardiac β3-AR expression, reversing the reduction of endothelial nitric oxide synthase (eNOS). *In vitro,* nebivolol treatment of palmitate-incubated H9C2 cells suppressed autophagy, restored mitochondrial biogenesis, leading to decreased mitochondrial reactive oxygen species (mtROS) generation, and suppressed NLRP3 inflammasome activation. Meanwhile the presence of shRNA against β3-AR or against eNOS deteriorated the protective effects of nebivolol. These data suggest the beneficial effect of nebivolol on myocardial lipotoxicity contributing to inhibiting NLRP3 inflammasome activation possibly via improved mitochondrial dysfunction.

The prevalence of juvenile obesity has increased dramatically worldwide in recent decades^1^,^2^. Obesity during adolescence has been found related with hyperlipidaemia, hyperglycaemia and hypertension. These adverse effects can disturb the homeostasis of cardiac energy metabolism, leading to myocardial inflammation. Myocardial inflammation is related with hypertrophy, fibrosis, apoptosis, and contractile dysfunction^3^. Emerging evidence indicates that not only infiltrating inflammatory cell induces local cardiac inflammation, but also cardiac cells, such as cardiac myocytes, fibroblasts, and endothelial cells, could likewise secrete pro-inflammatory cytokines and may thus aggravate cardiac dysfunction^4^. However, the mechanism underlying cardiac dysfunction induced by metabolic abnormalities remains unclear.

More recently, the Nod-like receptor (NLRs) is considered to have a critical role in detecting obesity-associated signals and initiating the inflammatory response^5^. Upon activation, some NLR proteins, including NLRP3, form multiprotein inflammasome complexes that cleave and activate caspase-1, leading to the secretion of Interleukin-1β (IL-1β) and Interleukin-18 (IL-18)^6^. It has been recently shown that the NLRP3 inflammasome is not restricted to immune cells, but also functional in non-immune cells, including myocytes and fibroblasts^7^,^8^. Inflammasome activation requires two signals, a priming signal that results in the transcription of IL-1β and IL-18, and a second signal that promotes indirect activation of the inflammasome, such as reactive oxygen species (ROS), ion or membrane perturbations, or extracellular ATP^9^,^10^. Noteworthy is that impaired mitochondrial turnover might be linked to mitochondrial defects and inflammasome activation^11^.

Although multiple randomised trials have shown that prevention of adolescent obesity reduces the occurrence of cardiovascular disease in adulthood, an effective potential therapy is missing. Nebivolol is a third-generation β-Adrenergic receptor (β-AR) blocker which has the highest β1-receptor affinity among β-blockers with vasodilator and antioxidant properties^12^. It also has advantages in improving lipid and glucose metabolism in obese individuals^13^. Our previous study demonstrated that nebivolol enhances mitochondrial biogenesis and increases the expression of antioxidant enzymes, such as manganese superoxide dismutase (MnSOD) and catalase, in adipocytes. These benefits are mediated by the eNOS/cyclic guanosine monophosphate (cGMP) -dependent pathway and are initiated by the activation of β3-AR receptors^14^. Other studies indicate that nebivolol has anti-inflammatory properties that could attenuate markers of inflammation^15^. However, whether nebivolol improves cardiac inflammation by inhibiting NLRP3 inflammasome activation has not been well-studied.

In this study, we examined the structural and functional properties of cardiac muscle in relation to inflammation features in a juvenile-adolescent animal model of diet-induced obesity. Furthermore, the rats were treated with nebivolol to determine the influence of nebivolol on mitochondrial quality, inflammasome components, oxidant load, and mitochondrial enzyme activities in cardiac tissue.

## Results

### Nebivolol improves cardiac function, lipid metabolism and systemic inflammatory

**Figure 1:**
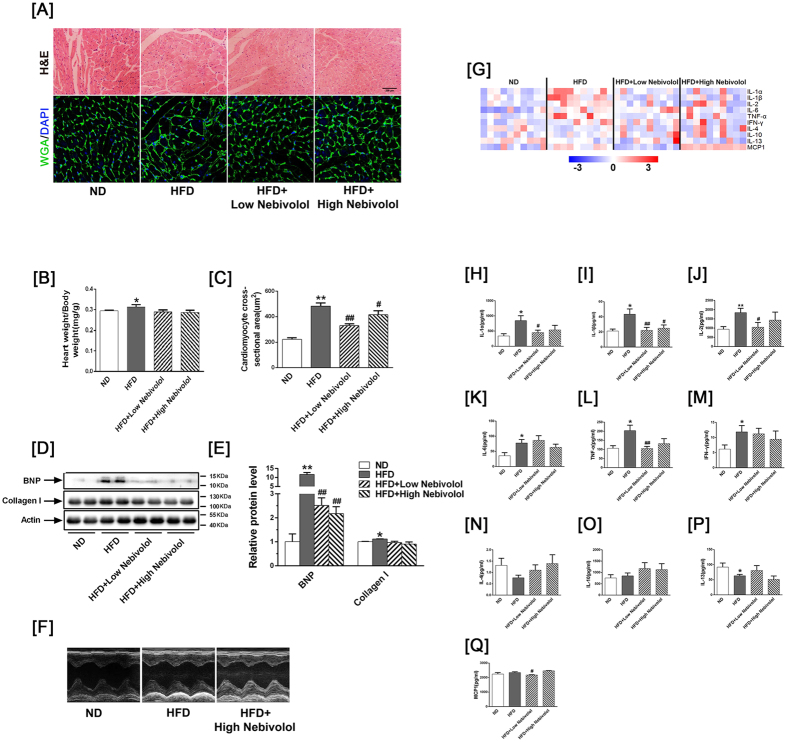
Nebivolol improves cardiac function, lipid metabolism and systemic inflammatory. (**A**) Representative images of cardiac sections stained with HE and WGA-FITC (nuclear staining with DAPI), scale bar = 200 μm. (**B**) Heart weight to body weight ratios (HW/BW; n=12). (**C**) Quantitative analyses of cardiomyocyte cross-sectional area (CSA). (**D**) Protein expression of BNP and Collagen-I. Left: representative western blot image; (**E**) Right: quantification of BNP and Collagen-I protein expression. (**F**) Echocardiographic M-mode images. (**G)** Heat map visualisation of the distribution of the different cytokine proteins in rat serum. In total, 10 cytokine proteins were detected by a fluorescent antibody array system: IL-1α, IL-1β, IL-2, IL-6, TNF-α, IFN-γ IFN I γ-4, IL-10, IL-13 and MCP-1. (**H–Q**) Each column represents values from an individual animal. Serum levels of IL-1α, IL-1β, IL-2, IL-6, TNF-α, IFN-γ, IL-4, IL-10, IL-13 and MCP-1. IL: interleukin; TNF-α: tumour necrosis factor alpha; IFN-γ: Interferon gamma; MCP-1: monocyte chemoattractant protein-1. The data are presented as means ± SEM (n = 12). *p < 0.05 vs. ND, **p < 0.01 vs. ND; ^#^*p* < 0.05 vs. HFD, ^##^*p* < 0.01 vs. HFD.

Body weight significantly increased in the HFD group compared to the ND group, whereas nebivolol reduced body weight in a dose-dependent manner. Systolic blood pressure (SBP) and heart rate (HR) were higher in the HFD groups than the ND groups; administration of nebivolol significantly decreased SBP and HR. Cardiac tissue analysis showed higher triglyceride and cholesterol levels in the HFD group, which were normalized by nebivolol supplement (Table 1). Histological analysis revealed that nebivolol treatment ameliorated HFD-induced cardiac hypertrophy, as measured by cardiomyocyte cross-sectional area (Fig. 1A–C). B-type natriuretic peptide (BNP) is a marker for hypertensive left ventricular hypertrophy. Western blotting revealed that the BNP and type 1 collagen level were elevated in the HFD compared to the ND group, and treatment with nebivolol decreased the BNP level (Fig. 1D,E).

As exemplified by the M-mode tracings in Fig. 1F and reported in Table S1, left ventricular end-diastolic diameter (LVESD) and left ventricular end-systolic diameter (LVEDD) were significantly increased in the HFD rats compared to the ND rats, while other echo-cardiographic parameters, such as left ventricular posterior wall (LVPW) showed a trend toward an increase in the HFD rats. Left ventricular fractional shortening (LVFS) was reduced in HFD rats compared to ND rats. Nebivolol administration prevented the increase in LVESD, LVEDD and LVPW and decrease in LVFS, but there was no statistically significant difference between the groups. These results demonstrate that obesity leads to impaired cardiac structure and function, which can be partly reversed by administration of nebivolol.

To investigate the expression pattern of pro-inflammatory and anti-inflammatory cytokines, we then used the Quantibody Rat-1 Array, which allows for measurement of the following serum cytokines: IL-1α, IL-1β, IL-2, IL-6, Tumor necrosis factor (TNF)-α, Interferon gamma (IFNγ), IL-4, IL-10, IL-13 and Monocyte chemoattractant protein-1 (MCP-1). The pro-inflammatory factor IL-1β significantly decreased in both high and low-dose nebivolol groups. IL-1α, IL-2, TNF-α and MCP-1 production significantly decreased following low-dose nebivolol administration. IL-1α, IL-2, TNF-α displayed a trend toward a decrease in high-dose nebivolol while IL-4 and IL-10 showed a trend toward an increase both in high and low-dose nebivolol administration, whereas there was no statistically significant difference between the groups. These results indicate that nebivolol can partly attenuate pro-inflammatory cytokine production and improve anti-inflammatory cytokine production (Fig. 1G–Q).

### Effects of nebivolol on parameters of cardiac oxidative stress and NLRP3 inflammasome activation

**Figure 2:**
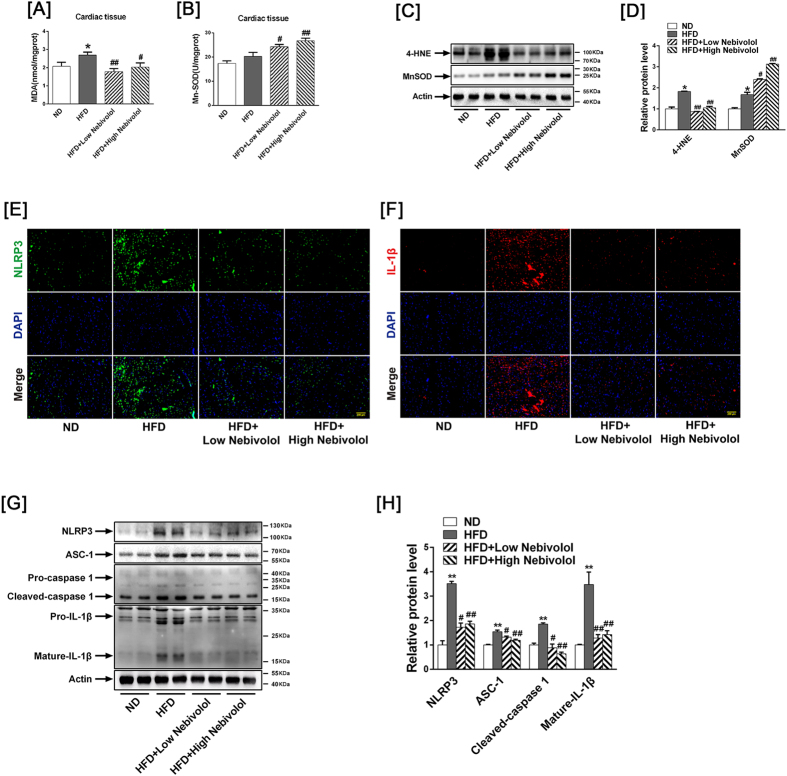
NLRP3 inflammasome activation in heart tissue. (**A**) MDA level in cardiac tissue. (**B**) MnSOD activity in cardiac tissue. (**C**) Western blot images of 4-HNE and MnSOD. (**D**) Quantitative band density analyses of 4-HNE and MnSOD. (**E**) Representative images of cardiac sections stained with NLRP3 (green) together with DNA staining with DAPI (blue), scale bar = 200 μm. (**F**) Representative images of cardiac sections stained with Il-1β (red) together with DNA staining with DAPI (blue), scale bar = 200 μm. (**G**) Representative Western blot images for NLRP3, ASC-1, Pro-caspase 1, Cleaved-caspase-1, Pro-IL-1β and Mature-IL-1β. (**H**) Quantitative band density analyses of NLRP3, ASC-1, caspase-1 and IL-1β. The results are presented as the fold increase in the ND group. The data are means ± SEM (n = 12). *p < 0.05 vs. ND, **p < 0.01 vs. ND; ^#^*p* < 0.05 vs. HFD, ^##^*p* < 0.01 vs. HFD. MDA: Malondialdehyde; Mn-SOD: Mn-superoxide dismutase.

Diet-induced obesity leads to metabolic heart disease characterised by increased oxidative stress. The malondialdehyde (MDA) level increased in the HFD rats compared to the ND rats, while nebivolol attenuated MDA content (Fig. 2A). Four-hydroxy-nonenal (4-HNE) is a major aldehyde produced during lipid peroxidation. As shown in Fig. 2C,D, 4-HNE-protein increased in heart tissues from HFD rats compared to ND rats, while nebivolol attenuated 4-HNE expression both in the low- and high-dose groups. Although there was no difference in MnSOD activities between the ND and HFD groups, nebivolol improved Mn-SOD activities, and both low- and high-dose nebivolol elevated MnSOD expression (Fig. 2B).

The NLRP3 inflammasome plays a substantial role in sensing obesity-associated inducers of caspase-1 activation and therefore regulates the magnitude of inflammation. To clarify the function of the NLRP3 inflammasome in cardiac tissue in the presence of obesity, cardiac sections were stained with NLRP3 and the pro-inflammatory factor Il-1β. Immunofluorescence revealed increased NLRP3 and Il-1β expression in heart tissues from HFD rats compared with ND rats, while nebivolol attenuated NLRP3 and Il-1β expression both in the low- and high-dose groups (Fig. 2E,F).Western blotting analysis revealed that the expression of NLRP3, apoptosis-associated speck-like protein (ASC)-1, cleaved-caspase 1 and mature IL-1β were elevated in the HFD compared to ND group. Treatment with nebivolol decreased NLRP3 inflammasome assembly, with evidence of attenuated NLRP3, ASC-1, cleaved-caspase-1 and mature IL-1β expression (Fig. 2G,H).

### Effects of nebivolol on cardiac mitochondrial dysfunction and dynamic

**Figure 3:**
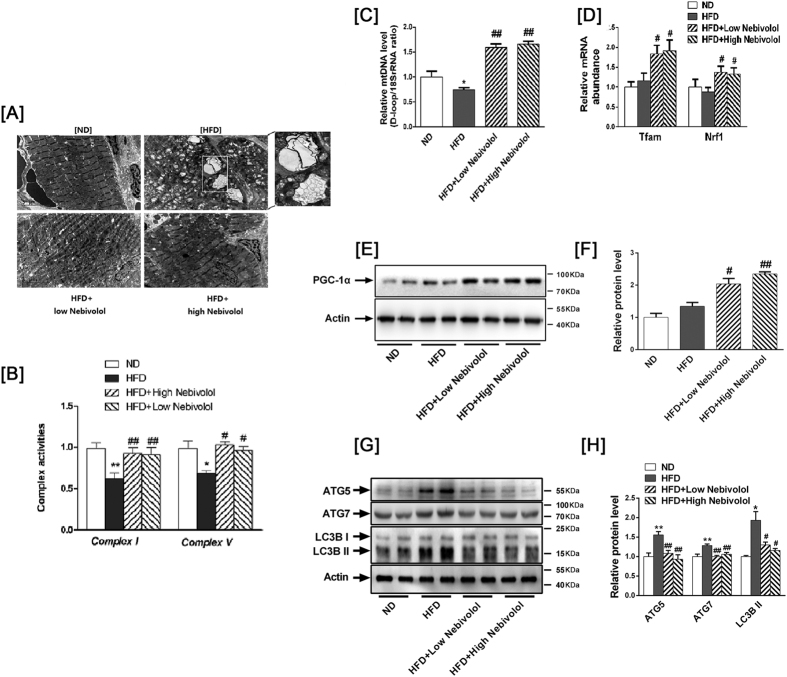
Mitochondrial dynamics in heart tissue. (**A**) Images from electron microscopy show clusters of grape-like mitochondrial networks in cardiac muscle (magnification 20,000). (**B**) Mitochondrial electron transport complexes I and V. (**C**) Mitochondrial DNA. The DNA content of mtDNA and the 18S rRNA gene were calculated, and the relative ratios of mtDNA content to 18S rRNA gene levels were determined. (**D**) mRNA abundance of Nrf1 and Tfam. (**E**) Protein expression of PGC-1α. Left: representative western blot image; (**F**) Right: quantification of PGC-1α protein expression. (**G**) Evidence of autophagy in heart tissue. Left: representative Western blot images for ATG5, ATG7 and LC3B. (**H**) Right: quantitative analyses of those target proteins. The results are presented as the fold increase in the ND group. The data are means ± SEM (n = 12). *p < 0.05 vs. ND, **p < 0.01 vs. ND; ^#^*p* < 0.05 vs. HFD, ^##^*p* < 0.01 vs. HFD.

As shown in Fig. 3A, the mitochondria were typically intact in the left ventricles from ND rats. The mitochondria were regular in shape, and compact with a high electron dense matrix. We also observed that HFD increased the number of autophagosomes in cardiac sections, and that the number of autophagosome was significantly lower in nebivolol treatment group. Our measurement showed that the activity of mitochondrial complex I and V (Fig. 3B) were significantly lower in HFD rats than in ND rats and the administration of nebivolol to the HFD rats remarkably increased the activity of complex I and complex V.

We then investigated the role of nebivolol on regulating mitochondrial quality control, including biogenesis and autophagy. Both low and high doses of nebivolol significantly increased the ratio of mitochondrial DNA (mtD-loop)/18S rRNA (Fig. 3C), PPARγ co-activator-1α (PGC-1α) protein level and the mRNA levels of nuclear respiratory factor 1(Nrf1) and mitochondrial transcription factor A (Tfam) (Fig. 3D–F), which are involved in regulating the expression of major mitochondrial proteins and mitochondrial DNA transcription and replication. Then, we investigated if increased mitochondrial biogenesis was accompanied by autophagy variation. The conversion of LC3-I to LC3-II, an indicator of autophagy activation, was assessed by immunoblot analysis using the LC3B antibody. Western blots showed that the LC3-II protein expression was elevated and accompanied by increases in the autophagy-related proteins ATG5 and ATG7 in the HFD rats, which could be reversed in a dose-dependent manner by nebivolol treatment (Fig. 3G–H). These results suggest that mitochondrial biogenesis is reduced while autophagy is up-regulated in high-fat diet-induced obesity. Nebivolol not only increased mitochondrial biogenesis but also decreased autophagy.

### β3AR- eNOS signalling pathway activation

**Figure 4:**
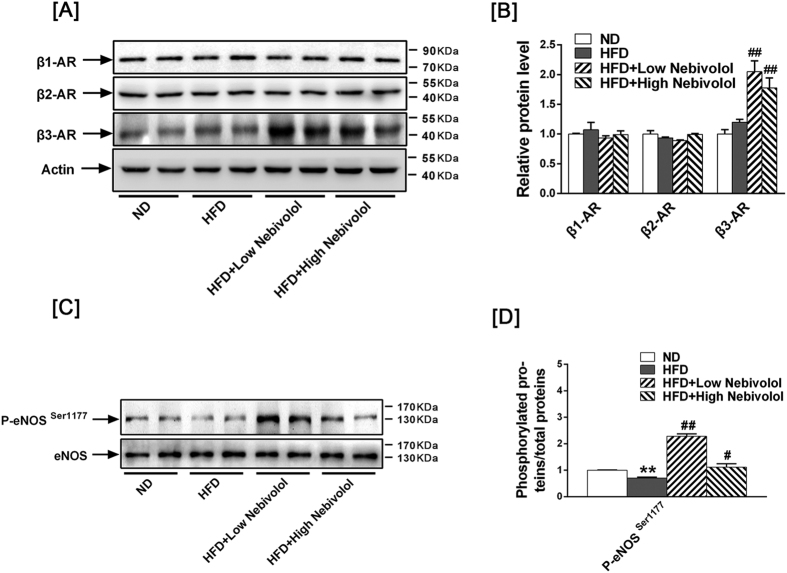
Signalling pathway activation in heart tissue. (**A**) Left: Representative Western blots images for β1-AR, β2-AR and β3-AR. (**B**) Right: quantitative analyses of those target proteins. The results are presented as the fold increase in the ND group. (**C**) Left: Representative Western blots images for p-eNOS^Ser1177^ and eNOS. (**D**) Right: quantitative analyses of p-eNOS^Ser1177^ and eNOS. The results are presented as the fold increase in the ND group. The results are presented as the fold increase in the ND group. The data are means ± SEM (n = 12). *p < 0.05 vs. ND, **p < 0.01 vs. ND, ^#^*p* < 0.05 vs. HFD, ^##^*p* < 0.01 vs. HFD.

To determine the role of β-AR in cardiac function, we investigated the expression of β1-AR, β2-AR and β3-AR by western blotting. Although there was no difference between ND and HFD group, both low- and high-dose of nebivolol increased β3AR expression (Fig. 4A,B), yet no changes in the expression of β1-AR and β2-AR were observed in any of the groups. In our previous study, we found that nebivolol induced mitochondrial biogenesis and antioxidant enzymes via the β3AR-eNOS signalling pathway. Consistently, both low- and high-dose nebivolol significantly induced eNOS activation (Fig. 4C,D).

### Reduction of β3-AR reduces the protective effects of nebivolol on mitochondrial dysfunction

**Figure 5:**
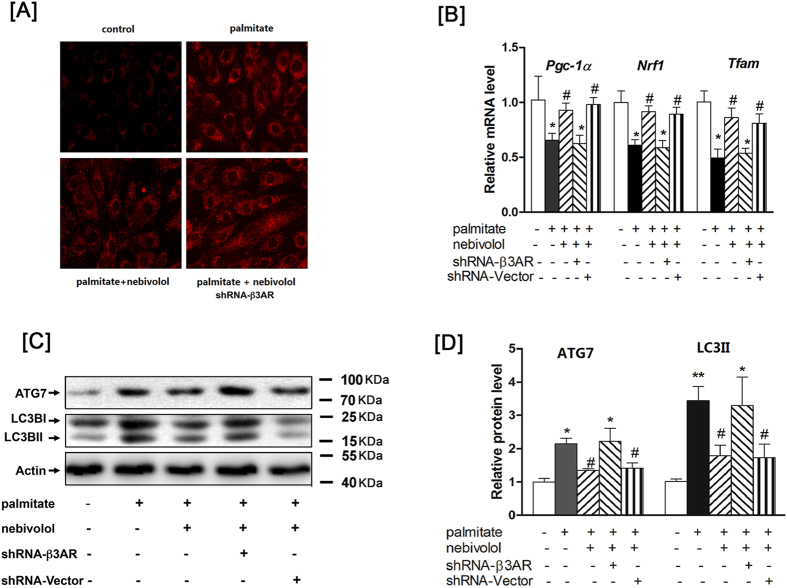
Reduction of β3-AR reduces the protective effects of nebivolol on mitochondrial dysfunction. (**A**) Blockade of β3AR suppresses the expression of palmitate-induced mitochondrial ROS production. (**B**) After infection with the shRNA, H9C2 were pre-treated with nebivolol (10  μM) for 1 h, followed by stimulation with palmitate for 16h. PGC-1a, NRF1 and TFAM mRNA levels were anyalyzed by qRT-PCR. (**C**) Cell lysates were analysed by western blotting with antibodies against β-actin, ATG7 and LC3B, representative Western blot images of β-actin, ATG7 and LC3B, (**D**) Quantification of protein expression. The results are presented relative to the values in the control cells. The data are the means ± SEM of four independent experiments. *p < 0.05 vs control, **p < 0.01 vs control, ^#^*p* < 0.05 vs palmitate, ^##^*p* < 0.01 vs palmitate.

To determine whether β3-AR is responsible for the effects of nebivolol on mitochondria, H9C2 cells were subjected to genetic blockade of β3-AR by shRNA. We confirmed that β3-AR expression was decreased by 80% after infection with β3-AR shRNA (data not shown). As shown in Fig. 5A, H9C2 cells were pre-incubated with nebivolol for 1 hr prior to a 4-hr treatment with palmitate, and the mitochondrial ROS production was measured with MitoTracker Red. Nebivolol significantly suppressed the palmitate-mediated ROS production, whereas the nebivolol-induced effects were completely reversed by knockdown of β3-AR in H9C2. We also examined the expression levels of genes involved in mitochondrial biogenesis (PGC1α, NRF1 and TFAM) and found that nebivolol completely reversed the palmitate-induced decrease in PGC1α, NRF1 and TFAM. Meanwhile, nebivolol reduced elevation of LC3II/LC3I level in H9C2 incubated with palmitate. In contrast, knockdown of β3-AR completely abrogated the enhanced levels of PGC-1α, NRF1 and TFAM while diminishing the inhibition of LC3II/LC3I level. Taken together, these data provide strong evidence that the protective effects of nebivolol on H9C2 mitochondrial dysfunction may be dependent on β3-AR (Fig. 5B–D).

### Nebivolol exposure improves mitochondrial quality through eNOS activation

**Figure 6:**
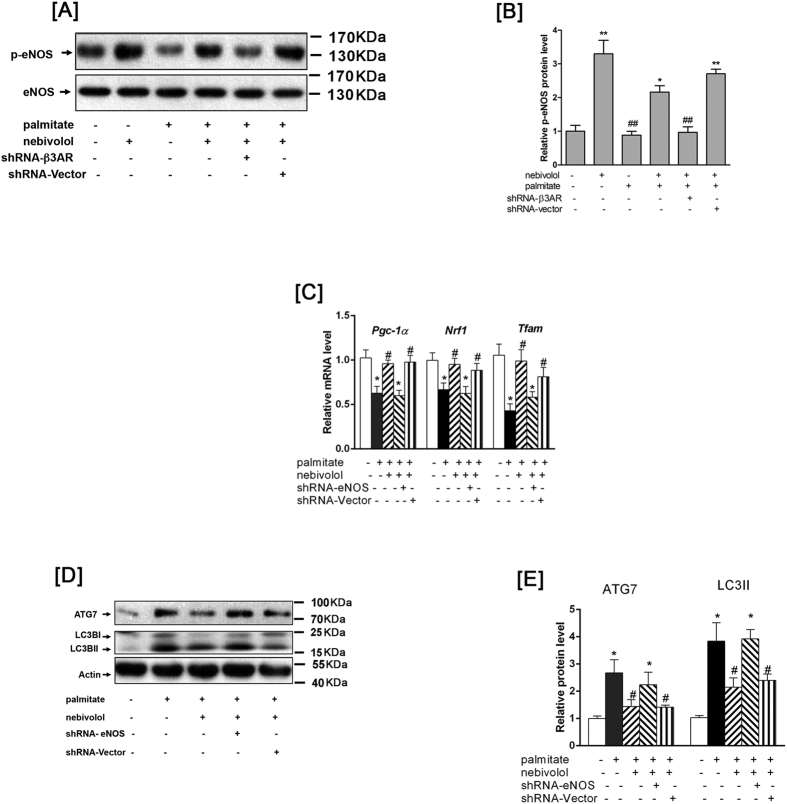
Nebivolol exposure improves mitochondrial quality through eNOS activation. (**A**) After infection with the shRNA against β3AR, then H9C2 cells were stimulated with nebivolol for 15 min. Cell lysates were analysed by western blotting with p-eNOS and eNOS. Representative Western blot images of p-eNOS and eNOS. (**B**) Quantification of protein expression. (**C**) After infection with the shRNA against eNOS, H9C2 were pre-treated with nebivolol (10  μM) for 1 h, followed by stimulation with palmitate for 16h. PGC-1a, NRF1 and TFAM mRNA levels were anyalyzed by qRT-PCR. (**D**) Cell lysates were analysed by western blotting with antibodies against β-actin, ATG7 and LC3B, representative Western blot images of β-actin, ATG7 and LC3B. (**E**) Quantification of protein expression. The results are presented relative to the values in the control cells. The data are the means ± SEM of four independent experiments. *p < 0.05 vs control, **p < 0.01 vs control, ^#^*p* < 0.05 vs palmitate, ^##^*p* < 0.01 vs palmitate.

Since nebivolol induced eNOS activation and increased mitochondrial biogenesis, we sought whether eNOS mediated the effects of nebivolol on mitochondrial biogenesis and functioned depending on β3AR. As shown in Fig. 6A,B, nebivolol elevated the palmitate-induced decrease in eNOS phosphorylation. Conversely, knockdown of β3AR reduced nebivolol-mediated eNOS activation in H9C2 cells. NO is demonstrated to be able to down-regulate autphagy; consistently, knockdown of eNOS resulted in decreased expression of PGC-1α, NRF1 and TFAM (Fig. 6C) while increasing LC3II/I expression in H9C2 cells (Fig. 6D,E). These results indicate that nebivolol exposure improves mitochondrial quality through eNOS activation.

### Nebivolol suppressed NLRP3 inflammasome activation through β3AR-eNOS pathway

**Figure 7:**
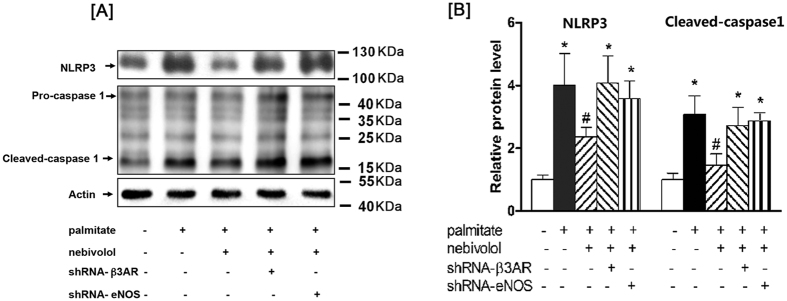
Nebivolol suppressed NLRP3 inflammasome activation through β3AR-eNOS pathway. (**A**) After infection with the shRNA against β3AR or eNOS, H9C2 were pre-treated with nebivolol (10  μM) for 1 h, followed by stimulation with palmitate for 16 h. Cell lysates were analysed by western blotting with antibodies against β-actin, NLRP3, and caspase-1, representative Western blot images of β-actin, NLRP3, Pro-caspase 1 and Cleaved-caspase-1. (**B**) Quantification of protein expression. The results are presented relative to the values in the control cells. The data are the means ± SEM of four independent experiments. *p < 0.05 vs control, **p < 0.01 vs control, ^#^*p* < 0.05 vs palmitate, ^##^*p* < 0.01 vs palmitate.

Then, we determined whether nebivolol suppressed NLRP3 inflammasome activation through β3AR-eNOS pathway. Nebivolol significantly suppressed palmitate-induced NLRP3 inflammasome activation in H9C2 cells. Treatment with β3AR or eNOS shRNA significantly attenuated the suppressive effect of nebivolol respectively, and the vector control had no effect on the H9C2 cells. These results indicate that nebivolol suppressed NLRP3 inflammasome activation through β3AR-eNOS pathway (Fig. 7A,B).

## Discussion

Juvenile obesity tends to induce pro-inflammatory cytokines by hyperlipidaemia, leading to persistent inflammation in myocardium, which contributes to cardiac dysfunction. In the present study, we found that treatment with nebivolol not only ameliorated the HFD-induced systemic inflammatory response but also alleviated cardiomyocyte inflammation by suppressing NLRP3 inflammasome activation. These improvements were associated with increased cardiac tissue mitochondrial function and network dynamics. Furthermore, blockade of the β3AR-eNOS pathway deteriorated the protective effects of nebivolol on mitochondrial function and inhibited the anti-inflammatory action of nebivolol.

Mitochondria dysfunction has been implicated in the cellular stress cascades leading to activation of NLRP3 inflammasome. In the present study, we demonstrated that high fat diet-related “lipotoxic danger signals”, such as triglycerides and total-cholesterol were markedly increased in DIO rats. The detrimental effect of hyperlipidaemia in cardiac tissue might induce an excessive mitochondrial load, as evidenced by an increase in the end products of lipid peroxidation, such as MDA and 4-HNE. Although MnSOD protein level was elevated in obese rats, possibly in an adaptative response to limit oxidative damage, the net effect on systemic oxidative stress was increased by an elevated MDA level and decreased MnSOD activity. Similarly, nebivolol significantly suppressed the palmitate-induced ROS generation in H9C2. These results suggest that nebivolol could reverse the imbalanced between prooxidants production and the cellular antioxidant defence system. In addition, we demostrated that obesity-induced mitochondrial dysfunction was involved in enhanced mitochondrial autophagy and reduced mitochondrial DNA content. Obesity is known to trigger cardiac hypertrophy contributing to sustained autophagy, which is a maladaptive feature of myocardial dysfunction. Recent studies have indicated that inhibition of excessive autophagy may contribute to attenuation of cardiomyocyte fibrosis and cardiac hypertrophy in obese rats^16^. Several lines of evidence suggest both excess and deficiency of autophagy can be detrimental to mitochondrial homeostasis^17^. In addition, studies have also shown that defective mitochondrial biogenesis in cardiomyocytes from young obese patients result in premature cardiac aging^18^. Thus, alterations in mitochondrial dynamics are associated with increased mitochondrial ROS production and tissue damage^19^.

Nebivolol is a third-generation β-AR blocker, which has high selectivity for blocking β1 and β3-agonistitic properties^20^. The β-adrenoceptors, belonging to the G protein-coupled receptor super family, play an essential role in cardiovascular function. Unlike β1- and β2-AR, which are mainly involved in mediating cardiac contraction, β3-AR regulates lipid metabolic disorders and activates both eNOS and neuronal NO synthases (nNOS), which may be associated with cardiac protective effects and vasodilatation^12^. As a partial agonist of β3-adrenergic receptor, nebivolol exhibits advantages over traditional beta-blockers with respect to glucose and lipid metabolism^21^,^22^. We have previously shown that nebivolol could promote adipocyte metabolism through increasing mitochondrial biogenesis^23^. The current study has shown that the high dose of nebivolol led to a decrease in body weight, consistent with previous report that nebivolol increases weight loss in patients with hypertension and diabetes^24^. Furthermore, to determine the receptor responsible for the effects of nebivolol on the mitochondrial networks, we used shRNA technology to knock down β3-AR expression in H9C2 cells. We found that the impact of nebivolol on promoting palmitate-induced decrease of mitochondrial content could be reversed by the genetic blockade of β3-AR. The change was paralleled by the mRNA levels of PGC-1a, NRF1 and TFAM, which positively regulate mitochondrial biogenesis in a β3-AR -dependent manner. We also showed that the ablation of β3-AR altered the effects of nebivolol on cardiomyocyte autophagy in response to lipid overload. These results suggest that β3-AR may be the primary receptor for mediating the nebivolol-induced alterations in mitochondrial dynamics and function in cardiomyocytes.

Furthermore, we found that phosphorylation of eNOS was decreased both in the obese rats and palmitate-treated H9C2 cells. Treatment with nebivolol significantly activated eNOS via phosphorylation at Ser 1177, whereas knockdown of β3AR reduced nebivolol-mediated eNOS activation and prevented nebivolol-induced expression of transcription factors involved in mitochondrial biogenesis, consistent with our previous reports that nebivolol induced mitochondrial biogenesis dependent on β3AR-eNOS-PGC1a pathway in adipocytes^23^.

Recent studies indicated that NO impaired autophagosome synthesis and endogenous NO by eNOS could inhibit autophagic flux as well^25^. Therefore, it seems that cardiac lipid-toxicity inhibited eNOS activity, which might induce autophagy at an early stage of autophagosome synthesis. Nebivolol inhibited palmintate-induced autophagic flux via eNOS activation, and blockade of eNOS by shRNA reversed nebivolol-mediated expression of autophagy related proteins in H9C2 cells. These results suggest that nebivolol improves mitochondrial quality via β3AR-eNOS in obese rats.

In the present study, the increase of NLRP3 inflammasome during adolescence was accompanied by cardiomyocyte hypertrophy in DIO rats, which indicated the importance of the NLRP3 inflammasome in the pathogenesis of obesity-realted cardiovascular diseases. NLRP3 forms multiprotein inflammasome complexes that cleave and activate caspase-1, which leads to the secretion of inflammatory cytokines. We observed elevations in a wide range of pro-inflammatory mediators, including IL-1α, IL-1β, IL-2, IL-6, TNF-α and IFN-γ. Nebivolol can partly attenuate pro-inflammatory cytokine production and improve anti-inflammatory cytokine production. However, nebivolol could not exhibit its anti-inflammatory effect in a dose-dependent manner. It is possible that since we evaluate the systemic inflammatory response in DIO rats treated with low-dose or high-dose nebivolol, pro-inflammatory factors were released by different immune cells and non- immune cells. Defective mitochondrial dynamic augments activation of the NLRP3 inflammasome has been recently described both in immune cells and non-immune cells from obese animals. In the present study, we found nebivolol ameliorates cardiac NLRP3 inflammasome activation accompanied by improving mitochondrial networks. To identify whether β3AR-eNOS pathway is linked to mitochondrial turnover and inflammasome activation, knockdown of β3AR or eNOS expression by shRNA attenuated the suppressive effect of nebivolol on NLRP3 inflammasome activation respectively. Hence, the suppressive effect of nebivolol on NLRP3 inflammasome activation is possibly via β3AR-eNOS pathway.

Our study provides evidence that administration of nebivolol in obese rats during adolescent improves cardiovascular risk factors by ameliorating HFD-induced systemic inflammation, attenuating NLRP3 inflammasome activation, reversing altered mitochondrial dynamic, restoring the antioxidant defence, and normalizing morphological and functional alterations related to hypertrophy and fibrosis. We suggest nebivolol markedly reduce myocardial NLRP3 inflammasome activation, contributing to improved mitochondrial quality via the β3AR/eNOS Pathway.

## Materials and Methods

### Materials

Anti-autophagy-related protein 5 (ATG5) and phospho-endothelial nitric oxide synthase (p-eNOS, Ser1177) antibodies were purchased from Cell Signaling Technology (Danvers, MA, USA). Antibodies against BNP, PGC-1α, IL-1β, β1-AR, β2-AR, β3-AR and ASC-1 were from Santa Cruz Biotechnology (Santa Cruz, CA, USA). Antibodies against ATG7, LC3B, Actin and NLRP3 were from Sigma (St. Louis, MO, USA). Anti-4-Hydroxy-2-noneal Michael Adducts (4-HNE), Caspase-1, MnSOD and catalase were from Millipore (Billerica, MA, USA). Peroxidase-conjugated rabbit anti-goat IgG, peroxidase-conjugated rabbit anti-mouse IgG, and peroxidase-conjugated goat anti-rabbit IgG were from Jackson ImmunoResearch (West Grove, PA, USA). The BCA™ protein assay kit and Pierce ECL Western blotting substrate were from Thermo Scientific (Rockford, IL, USA). Nebivolol was provided by Hanxiang Company (Wuhan, China). The reverse transcription system kit was from Promega (Mannheim, Germany) and HotStarTaq from Takara (Otsu, Shiga, Japan). *D-loop, Nrf1, Tfam* and *18S rRNA* primers were synthesised by Bioasia Biotech (Shanghai, China). Lentiviral shRNA β3-AR and shRNA against eNOS constructs were synthesised by Genechem Corporation (Shanghai, China).

### Animals

Male Sprague–Dawley (SD) rats were purchased from SLAC Laboratory Animal Co. Ltd. (Shanghai, China) and were housed in a temperature-controlled room with a 12-hour light/dark cycle. Water and rodent chow were provided *ad libitum*. All procedures were approved by the ethics review board of animal experiments at Shanghai Jiaotong University School of Medicine. All experiments involving the animals were conducted in conformance with the Guide for the Care and Use of Laboratory Animals published by the US National Institutes of Health (NIH Publication, 8th edition, 2011).

### Experimental protocol

A total of 60 four-week-old male rats were randomly divided into two groups: normal diet (ND, n = 15) and high-fat diet (HFD, n = 45). The ND group was fed a standard rat chow containing 11.2% fat, 55.5% carbohydrate, and 33.3% protein. The HFD animals received a high-fat diet containing 45.2% fat, 28.6% carbohydrate and 26.2% protein. After eight weeks, the HFD group was randomly divided into three groups with 12 rats each: high-fat diet (HFD), high-fat diet with low-dose nebivolol (HFD+ low nebivolol) and high-fat diet with high-dose nebivolol (HFD+ high nebivolol). The HFD+ low nebivolol group received nebivolol (5 mg/kg/d) and the HFD+ high nebivolol group received nebivolol (10 mg/kg/d) by gavage for four weeks, while the same volume of saline was given to the ND and HFD control groups. Food consumption, water intake and body weight were measured once a week. Heart rate (HR) and systolic blood pressure (BP) were measured using a Softron tail-cuff BP-98A non-invasive sphygmomanometer (Softron Incorporated, Tokyo, Japan).

### Cytokine proteins assessment

Blood samples were collected by cardiac puncture, and each blood sample was centrifuged at 1200 g for 10 min. The serum was removed and stored at –80 °C until analysis. Serum samples of rats were analysed using the Quantibody^®^ Rat Inflammation Array 1 (RayBiotech, Norcross, GA, USA) according to the manufacturer’s protocol. Axon scanner 4000 B with GenePix software was used to capture the fluorescent signals (green fluorescence, 532 nm excitation). Quantitative data analysis was performed using RayBio Q Analyzer software (RayBiotech, Inc.). The following 10 cytokines were detected: IL-1α, IL-1β, IL-2, IL-4, IL-6, IL-10, IL-13, MCP-1, IFN-γ and TNF-α. The detection limits for cytokines are displayed on the manufacturer’s website (http://www.raybiotech.com).

### Biochemical analysis

Cardiac tissue were finely minced and homogenized in ice-cold phosphate buffered saline (PBS). After centrifugation (1,000 g, 10 min), the supernatant was collected for analysis. Superoxide dismutase (SOD) activity, malondialdehyde (MDA) content, triglyceride (TG) and total cholesterol (TC) levels were analyzed using commercial clinical diagnosis kits (Jiancheng, Nanjing, China) according to the manufacturer’s instructions^26^.

### Histological analysis and immunofluorescence

After the experiment, cardiac tissues were fixed in 4% paraformaldehyde, embedded in paraffin, and then sectioned at 5 μm thickness. The specimens were stained with haematoxylin and eosin (H&E). Cardiomyocyte membranes were visualized using FITC-conjugated wheat germ agglutinin (WGA; Molecular Probes) followed by determination of the single cardiomyocyte cross-sectional area (CSA). Quantitative data from at least 100 cells were determined per group. The sections were then incubated with the antibodies for NLRP3 (1:300) and Il-1β (1:300), and further stained with Alexa Fluor 594-labelled anti-rabbit IgG and Alexa Fluor 488-labelled anti- rabbit IgG and nuclear staining with DAPI.

### Echocardiographic Measurement

Transthoracic echocardiographic study was performed on rats under isoflurane (2–2.5%) anaesthesia using a dynamically focused 15 MHz linear-array transducer (Visualsonics Vevo770 High-Resolution Imaging System, Canada). Imaging was performed at a 60° sector angle and 3 cm imaging depth. M-mode tracings were recorded from the short-axis view at the papillary muscle level of the left ventricle (LV).

### Transmission electron microscopy (TEM)

Fresh cardiac tissues were fixed in 2.5% (v/v) glutaraldehyde in phosphate-buffered saline, postfixed in 4% (w/v) osmium tetroxide, and embedded in Epon resin. Ultrathin sections (50–80 nm thick) were prepared, stained with lead citrate and uranyl acetate, and observed with a transmission electron microscope (CM 10; Philips, Eindhoven, the Netherlands)^27^.

### Isolation of mitochondria and mitochondrial complex I and complex V activity assay

Cardiac tissues were finely minced and homogenized in isolation buffer (in mmol/l): 70 sucrose, 210 mannitol, 1.0 EDTA-Na_**2**_, 50 Tris–HCl, pH 7.4 using a Potter-Elvejem homogenizer. The homogenate was transferred into a 2.0 ml Eppendorf tube and centrifuged at 1,300 × g for 3 min. The supernatant was carefully transferred into a clean 1.5 ml Eppendorf tube and centrifuged at 10,000 × g for 10 min. The pellet containing crude mitochondria was resuspended in isolation buffer^28^. NADH – CoQ oxidoreductase (complex I) activity was assayed by Kumar’s method^29^. Complex V activity was measured as oligomycin-sensitive Mg2 + -ATPase activity^30^.

### Western blotting

The soluble lysates (20 μg per lane) were separated by SDS-PAGE, transferred to a polyvinylidene fluoride membrane (Bio-Rad Laboratories; Hercules, USA) and blocked with 5% (w/v) nonfat milk/Tris-buffered saline Tween 20 (TBST) for 1 h at room temperature. The membranes were incubated overnight at 4 °C with primary antibodies directed against Αctin (1:10,000), BNP (1:1000), p-eNOS (1:1000), IL-1β (1:1000), MnSOD (1:8000), PGC-1α (1:1000), caspase-1 (1:1000), catalase (1:4000), NLRP3 (1:1000), LC3B (1:1000), ATG7 (1:1000), ASC-1(1:1000), β1-AR (1:1000), β2-AR (1:1000), β3-AR (1:1000) and 4-HNE (1:2000) in 5% (w/v) milk/TBST. The membranes were washed three times with TBST and then incubated with horseradish peroxidase-conjugated secondary antibody for 1 h at room temperature. Western blots were developed using electrochemoluminescence (Thermo Fisher Scientific Inc., Rockford, IL, USA) and quantified by scanning densitometry (Model 670 scanning densitometer, Bio-Rad Laboratories).

### Total DNA isolation and real-time PCR

Total DNA was extracted using the QIAamp DNA Mini kit (Qiagen, Hilden, Germany), and quantitative PCR was performed using mitochondrial DNA and genomic DNA-specific primers. The following primers were used:

***Mitochondrial D-loop** Forward:* 5′-AATCTACCATCCTCCGTG-3′;

*Reverse*: 5′-GACTAATGATTCTTCACCGT-3′;

***18S rRNA** Forward*: 5′-CGAACGTCTGCCCTATCAACTT-3′;

*Reverse: 5*′*-CTTGGATGTGGTAGCCGTTTCT-3*′.

The rat 18S rRNA gene served as the endogenous reference gene. A melting curve was performed to ensure specific amplification. The standard curve method was used for relative quantification. The ratio of the mitochondrial D-loop to 18S rRNA was then calculated. The results are presented as the fold of the ND group.

### RNA isolation and reverse transcription PCR

Total RNA was extracted from 30 mg of tissue using TRIzol reagent (Invitrogen, Carlsbad, USA) according to the manufacturer’s protocol. Samples of 2.0 μg total RNA were reverse transcribed into cDNA. Quantitative real-time PCR was performed using the 7300 Real-Time PCR System (Applied Biosystems, Carlsbad, USA) and SYBR Premix EX Taq TM (TaKaRa, Dalian, China). The primers were as follows:

***Nrf1*** Forward: 5′-TTACAGGGCGGTGAAATGAC-3′;

Reverse: 5′-GTTAAGGGCCATGGTGACAG-3′

***Tfam*** Forward: 5′-CCCTGGAAGCTTTCAGATACG-3′;

Reverse: 5′-AATTGCAGCCATGTGGAGG-3′;

***18S rRNA*** Forward: 5′-CGAACGTCTGCCCTATCAACTT-3′;

Reverse 5′-CTTGGATGTGGTAGCCGTTTCT-3′.

The rat 18S ribosomal RNA (rRNA) gene served as the endogenous reference gene. The evaluation of relative differences in PCR products among the treatment groups was performed using the ΔΔCT method. The reciprocal of 2CT (used CT as an exponent for the base 2) for each target gene was normalised using 18S rRNA. The results are presented as the fold of the ND group^31^.

### Cell culture and treatment

H9C2 cardiomyocytes were purchased from BioLeaf (Shanghai BioLeaf Biotech Co., Ltd, Shanghai, China). Cells were cultured at 37 °C in a humidified at mosphere with 95% air and 5% CO2. The cells were grown in Dulbecco’s modified Eagle’s medium (DMEM) containing 1000 mg/L glucose, 10% fetal calf serum, 2 mM glutamine, 100 IU/ml penicillin, and 100 mg/ml streptomycin. Cells were seeded on six-well plates and cultured in the growth medium for 24 h and then were pre-incubated with nebivolol (10 μmol/l) for 1 hr prior to incubated with or without palmitate (0.25 mmol/l) in serum starvation medium (DMEM containing 0.1% bovine serum albumin) for the indicated time periods.

### Infection of H9C2

A lentivirus-based shRNA expression system was used to knock down gene expression of β3-AR or eNOS and green fluorescent protein (GFP) constructs were used as the nontargeting shRNA controls. Cells were infected with the lentivirus at an m.o.i. of 25. The expression of β3-AR or eNOS was verified in H9C2 by immunoblotting.

### Mitochondrial sources of ROS measurement

MitoTracker Red CM-H2XROS (MT Red) was used to investigate whether the ROS generation inside mitochondria. After pretreatment with nebivolol for 1 h and then exposed to palmitate (0.25 mmol/l) for 4 h in the presence of nebivolol, H9C2 were incubated with 100 nmol/l MitoTracker Red for 30min at 37 °C. At the end of incubation, cells were washed for three times with cold PBS, and then images were analyzed with an Axiovert microscope (Carl Zeiss, Germany).

### Statistical analysis

The data are presented as the mean ± SEM. When the data are normally distributed, statistical significance was analysed using the unpaired Student’s t test or one-way ANOVA with Bonferroni’s test between the groups. If the data are not normally distributed, group comparison was performed using the Mann-Whitney test or Kruskal-Wallis test. The criterion for significance was set at *p* < 0.05.

## Additional Information

**How to cite this article**: Xie, Q. *et al*. Nebivolol Ameliorates Cardiac NLRP3 Inflammasome Activation in a Juvenile-Adolescent Animal Model of Diet-Induced Obesity. *Sci. Rep.* **6**, 34326; doi: 10.1038/srep34326 (2016).

## Supplementary Material

Supplementary Information

## Figures and Tables

**Table 1:** General characteristics of experimental rats (n = 12).

Characteristic	ND	HFD	HFD+ Low Nebivolol	HFD+ High Nebivolol
Final body weight (g)	583 ± 11	662 ± 14**	656 ± 25	606 ± 9#
Systolic blood pressure (mmHg)	142 ± 2	156 ± 2**	139 ± 2##	146 ± 1##
Heart rate (bpm)	310 ± 3	338 ± 8*	291 ± 5##	292 ± 3##
Triglyceride (nmol/mg ww)	2.65 ± 0.15	4.33 ± 0.14**	4.06 ± 0.21	3.60 ± 0.24#
Total cholesterol (nmol/mg ww)	2.33 ± 0.11	3.67 ± 0.11*	3.23 ± 0.12	3.03 ± 0.13#

Data are presented as mean± SEM. ^*^p < 0.05 vs. ND, ^**^p < 0.01 vs. ND. ^#^*p* < 0.05 vs. HFD, ^##^*p* < 0.01 vs. HFD.
