# Supplementary material for: Nebivolol Ameliorates Cardiac NLRP3 Inflammasome Activation in a Juvenile-Adolescent Animal Model of Diet-Induced Obesity
Source: Sci Rep. 2016 Sep 30;6:34326. doi: 10.1038/srep34326 (PMC5043271; doi:10.1038/srep34326)
Supplement: Supplementary Information [file srep34326-s1.doc]

**Nebivolol Ameliorates Cardiac NLRP3 Inflammasome Activation**

**in a Juvenile-Adolescent Animal Model of Diet-Induced Obesity**

*Qihai Xie^1,2§^, Tong Wei^1§^, Chenglin Huang^1^, Penghao Liu^1^, Mengwei Sun^3^,*

*Weili Shen^1^, Pingjin Gao^1^*

1. State Key Laboratory of Medical Genomics, Shanghai Key Laboratory of Hypertension, Department of Hypertension Ruijin Hospital, Shanghai Jiaotong University School of Medicine, Shanghai, China

2. Department of Cardiology, Shanghai Jiading District Central Hospital, Shanghai, China

3. Key Laboratory of State General Administration of Sport, Shanghai Research Institute of Sports Science, Shanghai, China

*Corresponding e-mail：[wlshen@sibs.ac.cn](mailto:wlshen@sibs.ac.cn)

*^§^*These authors contributed equally to this work.

**Table S1. Echocardiographic data (n=12)**

| **Characteristic** | **ND** | **HFD** | **HFD+High Nebivolol** |
| --- | --- | --- | --- |
| LVESD(mm) | 4.04 ± 0.06 | 4.44 ± 0.17* | 4.06 ± 0.22 |
| LVEDD(mm) | 8.00 ± 0.09 | 8.47 ± 0.11* | 7.77 ± 0.35 |
| LVPW(mm) | 1.69 ± 0.05 | 1.90 ± 0.07 | 1.76 ± 0.04 |
| LVFS(%) | 50.6 ± 1.79 | 46.2 ± 2.34 | 50.1 ± 3.38 |

LVESD: Left ventricular end-systolic dimension; LVEDD: Left ventricular end-diastolic dimension;

LVPW: Left ventricular posterior wall; LVFS: Left ventricular fractional shortening.

Data are presented as mean± SEM. **p* < 0.05 vs. ND.
